# Corticotropin-stimulated steroid profiles to predict shock development and mortality in sepsis: From the HYPRESS study

**DOI:** 10.1186/s13054-022-04224-5

**Published:** 2022-11-07

**Authors:** Josef Briegel, Patrick Möhnle, Didier Keh, Johanna M. Lindner, Anna C. Vetter, Holger Bogatsch, Dorothea Lange, Sandra Frank, Ludwig C. Hinske, Djillali Annane, Michael Vogeser, SepNet Critical Care Trials Group, Michael Bauer, Thorsten Brenner, Patrick Meybohm, Markus Weigand, Matthias Gründling, Markus Löffler, Michael Kiehntopf, Frank Bloos, Gunnar Elke, Melanie Meersch-Dini, Christian Putensen, Achim Kaasch, Stefan Kluge

**Affiliations:** 1 Department of Anesthesiology, University Hospital, LMU Munich, Munich, Germany; 2 Department of Transfusion Medicine, Cellular Therapeutics and Hemostaseology, Department of Anesthesiology, LMU Munich, Munich, Germany; 3 Department of Anesthesiology and Intensive Care Medicine, Charité University Hospital, Berlin, Germany; 4 Institute of Laboratory Medicine, University Hospital, LMU Munich, Munich, Germany; 5 Clinical Trial Centre, Leipzig University, Leipzig, Germany; 6 Institute for Biomedical Information Processing, Biometry and Epidemiology, LMU Munich, Munich, Germany; 7 General ICU - Raymond Poincaré Hospital, Assistance Publique - Hôpitaux de Paris (APHP), Paris, France; 8 Klinik Für Anästhesiologie, Klinikum der Ludwig-Maximilians-Universität (LMU), Marchioninistrasse 15, E 81377 Munich, Germany

**Keywords:** Sepsis, Shock, Septic, Steroids, Mass spectrometry, Corticosterone, Hydrocortisone, Hospital mortality

## Abstract

**Rationale:**

Steroid profiles in combination with a corticotropin stimulation test provide information about steroidogenesis and its functional reserves in critically ill patients.

**Objectives:**

We investigated whether steroid profiles before and after corticotropin stimulation can predict the risk of in-hospital death in sepsis.

**Methods:**

An exploratory data analysis of a double blind, randomized trial in sepsis (HYPRESS [HYdrocortisone for PRevention of Septic Shock]) was performed. The trial included adult patients with sepsis who were not in shock and were randomly assigned to placebo or hydrocortisone treatment. Corticotropin tests were performed in patients prior to randomization and in healthy subjects. Cortisol and precursors of glucocorticoids (17-OH-progesterone, 11-desoxycortisol) and mineralocorticoids (11-desoxycorticosterone, corticosterone) were analyzed using the multi-analyte stable isotope dilution method (LC–MS/MS). Measurement results from healthy subjects were used to determine reference ranges, and those from placebo patients to predict in-hospital mortality.

**Measurements and main results:**

Corticotropin tests from 180 patients and 20 volunteers were included. Compared to healthy subjects, patients with sepsis had elevated levels of 11-desoxycorticosterone and 11-desoxycortisol, consistent with activation of both glucocorticoid and mineralocorticoid pathways. After stimulation with corticotropin, the cortisol response was subnormal in 12% and the corticosterone response in 50% of sepsis patients. In placebo patients (*n* = 90), a corticotropin-stimulated cortisol-to-corticosterone ratio > 32.2 predicted in-hospital mortality (AUC 0.8 CI 0.70–0.88; sensitivity 83%; and specificity 78%). This ratio also predicted risk of shock development and 90-day mortality.

**Conclusions:**

In this exploratory analysis, we found that in sepsis mineralocorticoid steroidogenesis was more frequently impaired than glucocorticoid steroidogenesis. The corticotropin-stimulated cortisol-to-corticosterone ratio predicts the risk of in-hospital death.

*Trial registration* Clinical trial registered with www.clinicaltrials.gov Identifier: NCT00670254. Registered 1 May 2008, https://clinicaltrials.gov/ct2/show/NCT00670254.

## Introduction

Adequate activation of the hypothalamic–pituitary–adrenal axis (HPA axis) is paramount to respond appropriately to severe infections and sepsis [1]. The corticotropin test has long been proposed to assess the adrenocortical reserve and to prognosticate mortality in septic shock [2]. In sepsis, the changes in steroid metabolism can affect the function of the HPA axis in very complex and time-dependent ways [3]. Several studies demonstrated an inflammatory mediator-mediated decreased corticotropin synthesis, prolonged metabolism of corticosteroids, increased volume of distribution for corticosteroids, altered steroid delivery to the tissues, and decreased tissue sensitivity to steroids in sepsis [1, 3–6].

Many of the studies on sepsis focus on cortisol as the main active corticosteroid in humans. Van den Berghe and co-workers showed that cortisol levels in sepsis depend on corticotropin-driven adrenal secretion only in the hyperacute phase, thereafter more on delayed cortisol degradation, an increased volume of distribution, and the extent of peripheral metabolism, i.e., inactivation to cortisone or reactivation from cortisone via 11 beta-hydroxysteroid dehydrogenase (11β-HSD) [3, 5–10]. Cortisol, as the classic readout of the corticotropin test, can therefore be altered by various conditions, in particular by an increased volume of distribution, as typically occurs in sepsis [3, 5, 11].

It is known from long-standing studies that corticotropin not only stimulates the synthesis and release of cortisol, but also activates the mineralocorticoid pathway. Although the hormone aldosterone is predominantly controlled by renin and angiotensin, the synthesis of its precursor corticosterone is exclusively triggered by corticotropin [12, 13]. It has been suggested that the adequate amount of corticosterone may play a central role at this interface of two endocrine regulatory circuits, especially in critical illness [14].

In a study in healthy volunteers, we showed that serum corticosterone exhibits a highly dynamic response after stimulation with corticotropin and appears to be a very sensitive biochemical marker of stress [15]. Therefore, the corticotropin-stimulated response of corticosterone, the precursor of aldosterone, may be of interest in critically ill patients, especially in patients at the onset of sepsis.

We hypothesized that corticotropin-stimulated steroid profiles could predict various endpoints typically chosen in sepsis studies. For this reason, we undertook an exploratory analysis of stimulated steroid profiles in patients with sepsis not being in shock in comparison with healthy individuals. In particular, we investigated metabolites of glucocorticoid and mineralocorticoid pathways in predicting the in-hospital mortality. For this purpose, we used data and samples from healthy volunteers and the HYPRESS trial, from which the central laboratory of the LMU Munich provided the measured values of the corticotropin tests [16].

## Methods

**Fig. 1:**
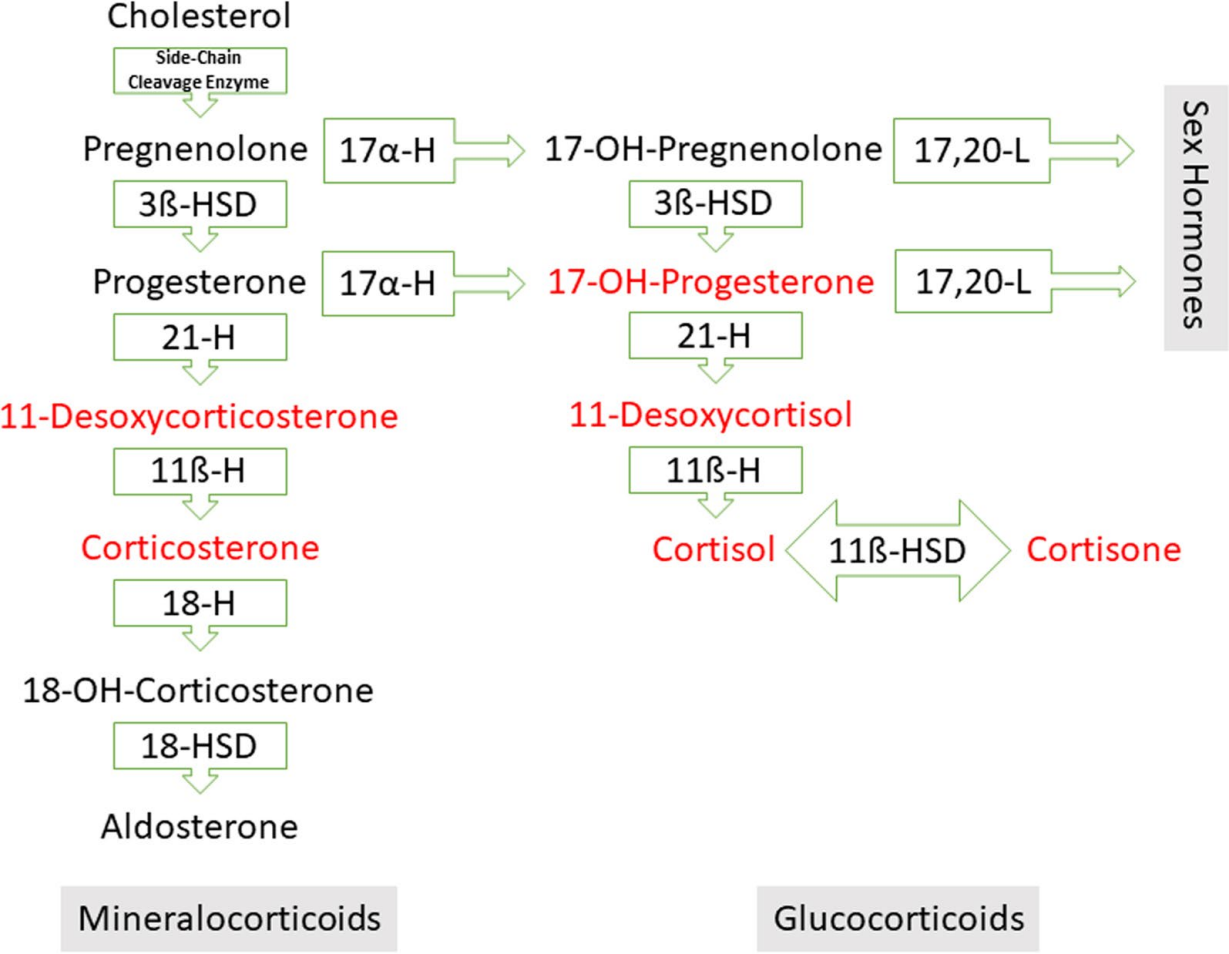
Outline of the mineralocorticoid and glucocorticoid pathways in steroidogenesis. Corticotropin boosts steroidogenesis including precursors of mineralocorticoids. Aldosterone secretion is predominantly controlled by the renin-angiotensin system. The analyzed corticosteroids are shown in red. HSD: Hydroxysteroid dehydrogenase; H: hydrolase; and L: lyase

The HYdrocortisone for PRevention of Septic Shock (HYPRESS) study was an investigator-initiated, multicenter, placebo-controlled, double-blind RCT supported by the German Federal Ministry of Education and Research [16]. The responsible ethics committees of all 34 participating sites approved the protocol. Corticotropin testing was part of the protocol prior to randomization with samples taken before and 60 min after administration of 250 μg of corticotropin (Synacthen®) [16]. To avoid possible inter-assay variations, all measurements of cortisol were done in one laboratory at the Department of Laboratory Medicine, Klinikum der Ludwig-Maximilians-Universität, Munich, Germany [17]. Multi-analyte, stable isotope-dilution LC–MS/MS method was used for measurement of cortisol. In addition to cortisol, the simultaneous measurement of glucocorticoid and mineralocorticoid precursors and metabolites (i.e., 11-desoxycorticosterone, corticosterone, 17-OH-progesterone, 11-desoxycortisol, and cortisone, see Fig. 1) was performed using this reference method [18]. The steroid profile of healthy subjects before and after corticotropin stimulation served as a control and to determine the reference range of the steroids studied. The ethical committee of LMU Munich (No. 84-15) approved this part of the study. Further details including the LC-MS/MS method can be found in [15].

The sepsis study cohort included patients with severe sepsis without shock who met the appropriate inclusion criteria of the HYPRESS trial [16]. For analysis of steroid profiles, patients were excluded if they had received steroids or etomidate, which are known to interfere with adrenal steroidogenesis, in the days before enrollment in the study. The clinical data of the HYPRESS trial were submitted in pseudonymized form by the Clinical Trial Centre (ZKS) Leipzig (H. Bogatsch). These included baseline variables like age, sex, SAPS and SOFA score, lactate, type of underlying infection, type of organ dysfunction, and outcome variables such as in-hospital mortality, mortality at different time points (day 28, 90, 180), and development of septic shock within 14 days (primary endpoint of the HYPRESS trial).

The aim of this exploratory analysis was to compare the activation of glucocorticoid and mineralocorticoid pathways in healthy individuals vs. patients in sepsis without shock. In addition to the classical readout of the corticotropin test, additional steroids and precursors were investigated simultaneously with regard to their prognostic value at baseline and 60 min after stimulation with corticotropin. Since we could previously show that the dynamics of serum corticosterone after stimulation with corticotropin seems to be a very sensitive biochemical stress marker, the prognostic value of this marker was analyzed in combination with cortisol, the classical readout of the corticotropin test [15]. The outcome analysis of in-hospital mortality was performed using only the steroid profiles of patients in the placebo group, with the assumption that treatment with hydrocortisone could alter a patient's individual outcome in either direction (improved or worsened survival).

### Statistical analysis

The cohorts for descriptive analysis of steroid profiles consisted of patients with sepsis from both treatment groups (placebo and hydrocortisone) and healthy individuals. The sepsis cohort for the outcome analysis came exclusively from the placebo group. We compared steroid profiles in patients who were discharged from hospital or who died in hospital with steroid profiles in healthy individuals. Data of the groups are shown as box-and-whisker plots with median, 1^st^ and 3^rd^ quartiles, range, and possible extreme values and were analyzed by Kruskal–Wallis test with Conover post hoc analysis. In order to compare the diagnostic performance of the analyzed steroids or their precursors, receiver operating characteristic (ROC) curves with the outcome variable in-hospital mortality were used. Kaplan–Meier survival plots were constructed to examine stratification by prognostic marker cutoffs for development of septic shock and 90-day survival in both groups (placebo and hydrocortisone). The statistical analysis was done using MedCalc Statistical Software Version 20.0008 (MedCalc Software BV, Belgium; https://www.medcalc.org; 2021).

The study plan and preliminary results of this exploratory analysis were presented at two internal meetings of the SepNet Critical Care Trials Group, which conducted the HYPRESS Trial.

## Results

Corticotropin tests were performed in 206 patients with sepsis without shock [16]. Patients who had received etomidate (*n* = 13) or exogenous steroids (*n* = 12) prior to enrollment were excluded from this analysis. A patient's corticotropin test, which showed no changes in all analytes, was also excluded due to a suspected error in the test procedure. The cohort under investigation consisted of 180 patients with severe sepsis (67 females) and 20 healthy volunteers (10 females). The characteristics of the cohorts are shown in Table 1.

**Table 1:** Characteristics of healthy individuals and patients with severe sepsis

Characteristic	Healthy Ind	Sepsis Placebo	Sepsis Steroid
Male, No. (%)/total No	10/20 (50)	53/90 (59)	60/90 (67)
Age, mean (SD), y	33.1 (9.9)*	62.9 (15.7)	64.3 (14.7)
Focus of primary infection, No./total No. (%)			
Known focus	n.a	76/90 (84)	83/90 (92)
Pneumonia	n.a	36/90 (40)	28/90 (31)
Intraabdominal infection	n.a	2/90 (2.2)	6/90 (6.7)
Urogenital	n.a	12/90 (13)	14/90 (16)
Other infection	n.a	26/90 (29)	35/90 (39)
Organ dysfunction, No./total No. (%)			
Central nervous system	n.a	21/90 (23)	17/90 (19)
Respiratory	n.a	59/90 (66)	59/90 (66)
Renal	n.a	41/90 (46)	41/90 (46)
Microcirculatory	n.a	14/90 (16)	24/90 (27)
Coagulation	n.a	15/90 (16)	22/90 (24)
SOFA score, mean (SD)	n.a	6.0 (2.3)	6.4 (2.5)
SAPS II score, mean (SD)	n.a	50.9 (9.7)	52.7 (14.7)
Outcome			
Septic Shock, No./total No. (%)	n.a	18/90 (20)	18/90 (20)
Mortality, No./total No. (%) in-hospital	n.a	12/90 (13)	10/89 (11)
Mortality, No./total No. (%) 28 day	n.a	8/88 (9.1)	8/90 (8.9)
Mortality, No./total No. (%) 90 day	n.a	15/86 (17)	17/89 (19)

*p < 0.05 Kruskal–Wallis test (*H* test) and post hoc test Conover or Mann–Whitney *U* tests, respectively

Sepsis patients were older than the cohort of healthy individuals was. In the sepsis cohort, 90 patients were treated with hydrocortisone and 90 patients with placebo after the corticotropin test. There was no significant difference in the clinical data and the steroid profiles between placebo and hydrocortisone allocation. Thirty-six patients developed septic shock until day 14 and 16 patients died until day 28. In-hospital mortality was reported to be 12% in 179 of 180 patients under study (Table 1).

In healthy individuals, the steroid profile after corticotropin stimulation showed a variable and highly dynamic response in all steroids and precursors under analysis (see details in Fig. 1). In particular, the response of serum corticosterone concentration to corticotropin was highly dynamic with a 14.3-fold increase in healthy subjects (Table 2) [15]. No differences regarding gender were observed with the exception of 17-OH progesterone being lower at baseline but not after corticotropin in females.

**Table 2:** Corticotropin-stimulated steroid profiles in placebo patients with sepsis and healthy individuals: peak concentrations and dynamic response to corticotropin

	Sepsis Placebo Non-survivors (*n* = 12)			Sepsis Placebo survivors (*n* = 78)			Healthy volunteers (*n* = 20)		
	Median [µg/L]	Quartiles [µg/L]	x-fold increase	Median [µg/L]	Quartiles [µg/L]	x-fold increase	Median [µg/L]	Quartiles [µg/L]	x-fold increase
11-Desoxycorticosterone	0.3	0.2–0.8	4.2	0.4*	0.2–0.9	6.8	0.2	0.1–0.3	8.1
11-Desoxycortisol	4.6*	1.2–21.4	4.2	3.0*	1.6–6.3	6.2*	1.0	0.5–1.1	3,8
17-OH-Progesterone	2.2	0.8–3.4	4.3	1.8*	1.3–3.1	6.1*	1.4	1.0–2.0	1.9
Corticosterone	8.2*#	3.0–17.8	4.8*#	18.4* +	10.3–25.2	7.8 +	30.3	27.4–34.4	14.3
Cortisol	328.2*	222.3–472.9	1.5	352.1*	281.6–447.1	1.9	258.6	244.9–269.3	2.1
Cortisone	16.9	15.1–21.3	1.2	17.6	13.7–22.2	1.1	19.5	17.6–20.5	1.0

The data are presented as medians, quartiles, and x-fold increases. * Different from healthy volunteers (*p* < 0.05); # different from sepsis survivors (*p* < 0.05); and + different from sepsis non-survivors (*p* < 0.05)

**Fig. 2:**
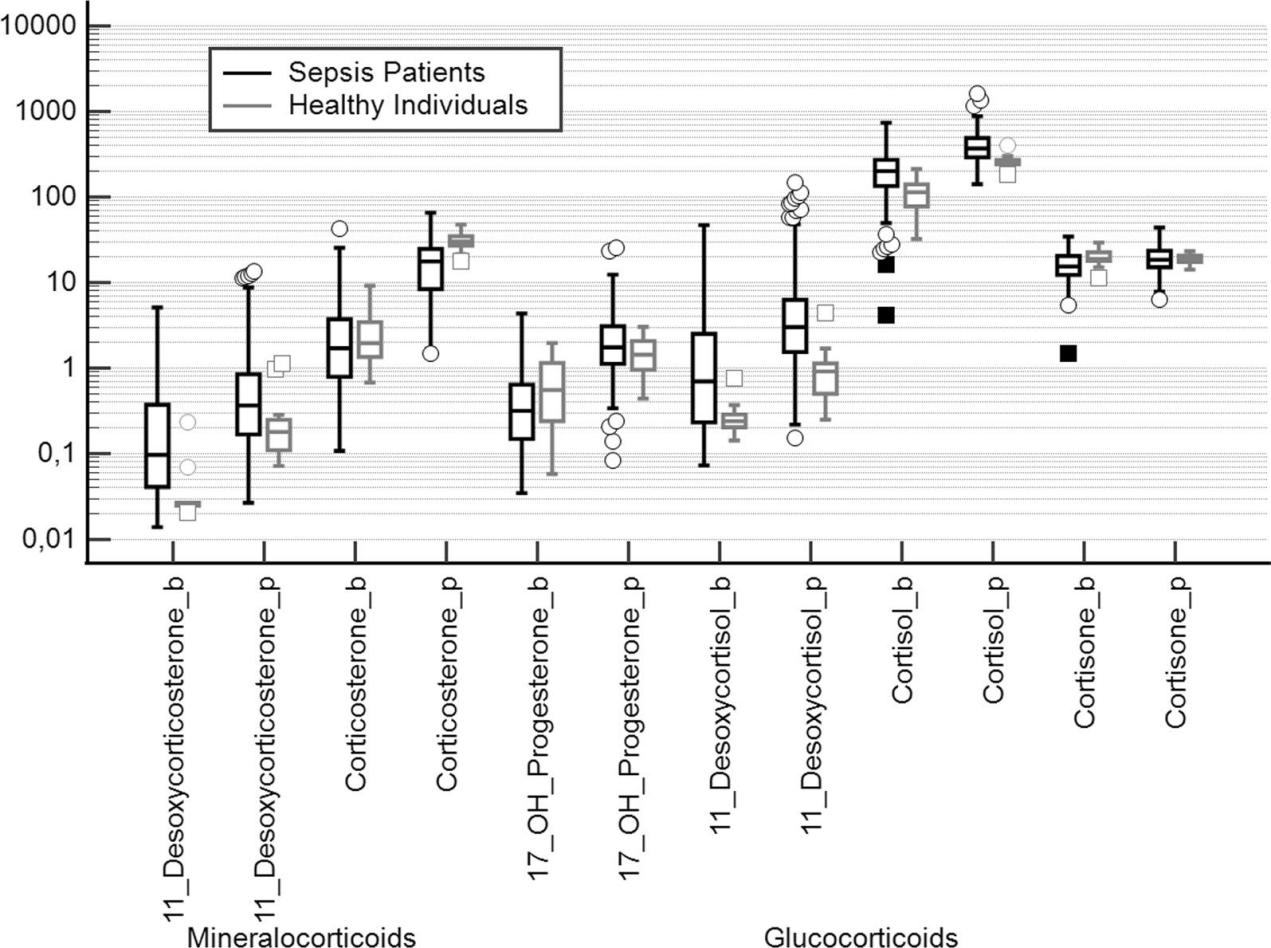
Steroid profile at baseline (b) and 60 min (p) after corticotropin (Synacthen®) in severe sepsis without shock and healthy individuals. 11-Desoxycorticosterone at baseline and after stimulation was elevated in severe sepsis compared to healthy individuals (*p* < 0.01 Mann–Whitney test). Corticosterone at baseline did not differ between the groups. After stimulation with corticotropin (Synacthen®), corticosterone was significantly lower in sepsis (*p* < 0.0001). No differences were found for 17-OH-Progesterone (b and p). 11-Desoxycortisol (b and p) and cortisol (b and p) were significantly elevated in sepsis patients compared to healthy individuals indicating a shift toward cortisol synthesis in severe sepsis (all *p* < 0.0001). Cortisone at baseline was significantly lower in sepsis (*p* < 0.01) but not after stimulation with corticotropin. In severe sepsis, steroid profiling in combination with corticotropin testing showed activation of the glucocorticoid pathway and, in the mineralocorticoid pathway, an attenuated corticosterone biosynthesis despite high concentrations of its precursor

Compared to healthy individuals, patients with sepsis showed different steroid profiles. At baseline, there were significantly increased levels of 11-desoxycorticosterone, 11-desoxycortisol, and cortisol but not corticosterone. Compared to healthy subjects, corticosterone at baseline did not differ, and cortisone concentrations were significantly lower. After stimulation with corticotropin, patients with sepsis showed equal levels of the precursor 17-OH progesterone and significantly higher levels of 11-desoxycortiosterone, 11-desoxycortisol, and cortisol compared to healthy individuals. The increase in corticosterone, however, was significantly lower in patients with sepsis compared to healthy individuals (Fig. 2).

Our comparisons of healthy individuals and patients with severe sepsis showed that 50% of patients with sepsis (not in shock!) had a subnormal increase in corticosterone (i.e., delta-corticosterone < 17 µg/L, lower limit in healthy subjects) to corticotropin, whereas only 12% of the same cohort had a subnormal cortisol response (i.e., < 60 µg/L, lower limit in healthy subjects).

**Fig. 3:**
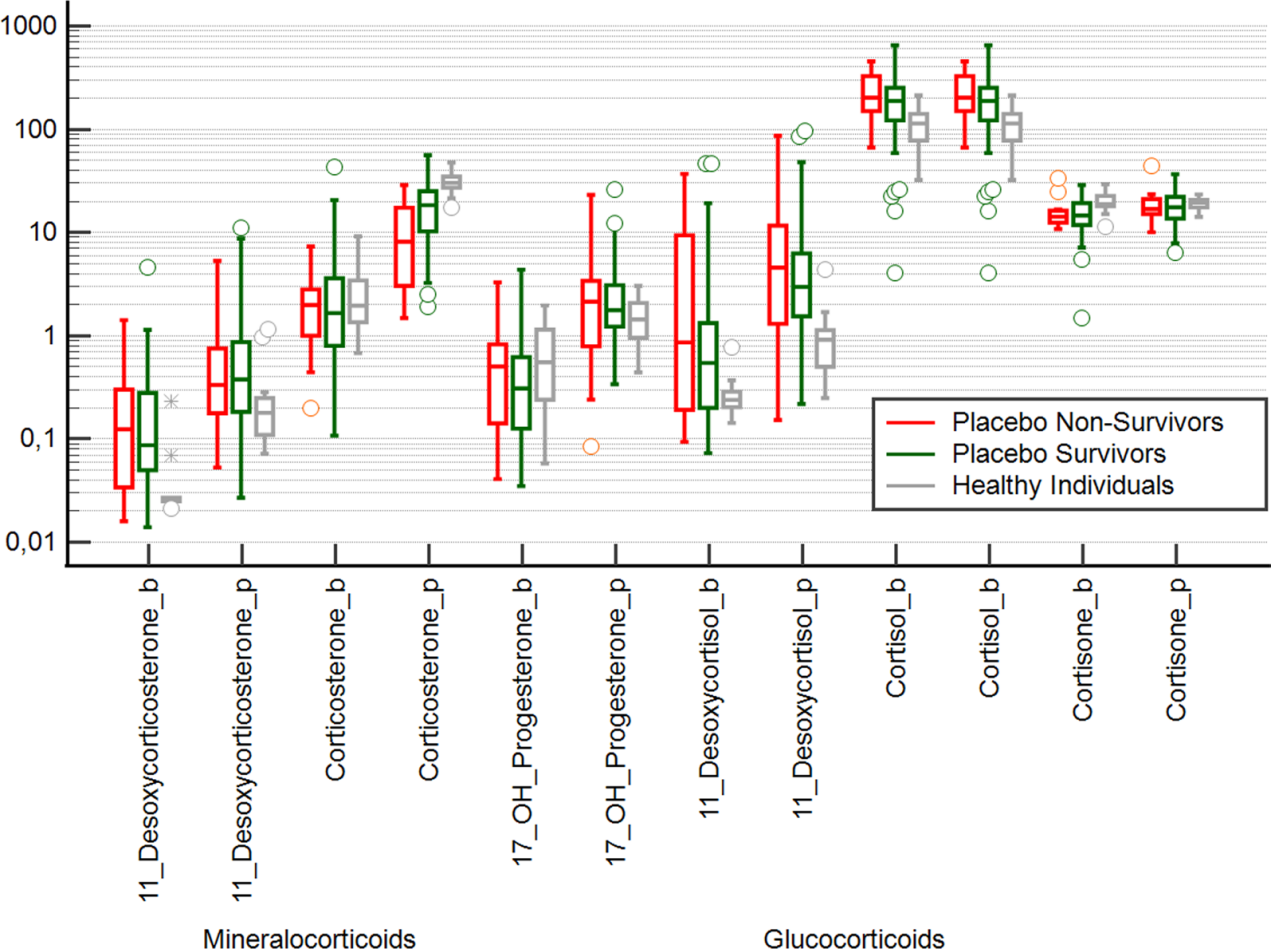
Steroid profile in placebo patients with sepsis and healthy individuals at baseline (b) and 60 min (p) after corticotropin (Synacthen®). Again, there is a significant increase in 11-desoxycorticosterone and 11-desoxycortisol in sepsis, consistent with activation of both the mineralocorticoid axis and the glucocorticoid axis. This results in significantly elevated cortisol levels, but not elevated corticosterone levels at baseline. After stimulation with corticotropin, corticosterone was significantly lower in patients who died in hospital compared to patients who survived sepsis and compared to healthy individuals (Kruskal–Wallis test *p* < 0.0001; Conover post hoc analysis: significant differences between all groups). 17-OH-Progesterone prior and after corticotropin was not different between the three groups. Cortisone at baseline was significantly lower in sepsis (*p* < 0.01) but not after stimulation with corticotropin

The outcome analysis (in-hospital mortality) of the placebo group showed that non-surviving sepsis patients had significantly lower increases in corticosterone than surviving sepsis patients, suggesting a greater impairment of mineralocorticoid metabolism in non-survivors. It should be noted that the corticotropin-induced increase in 11-desoxycorticosterone did not result in a corresponding increase in corticosterone, indicating an impediment to this synthetic pathway of mineralocorticoid steroidogenesis (Table 2 and Fig. 3).

Corticosterone after corticotropin was found to have a good prognostic ability for in-hospital mortality. In the ROC analysis, an area under the curve of 0.73 (CI 0.62–0.82) was found with a sensitivity of 67% and a specificity of 80%. The corresponding criterion for a fatal outcome was a stimulated corticosterone level less than or equal to 8.5 [µg/L].

**Fig. 4:**
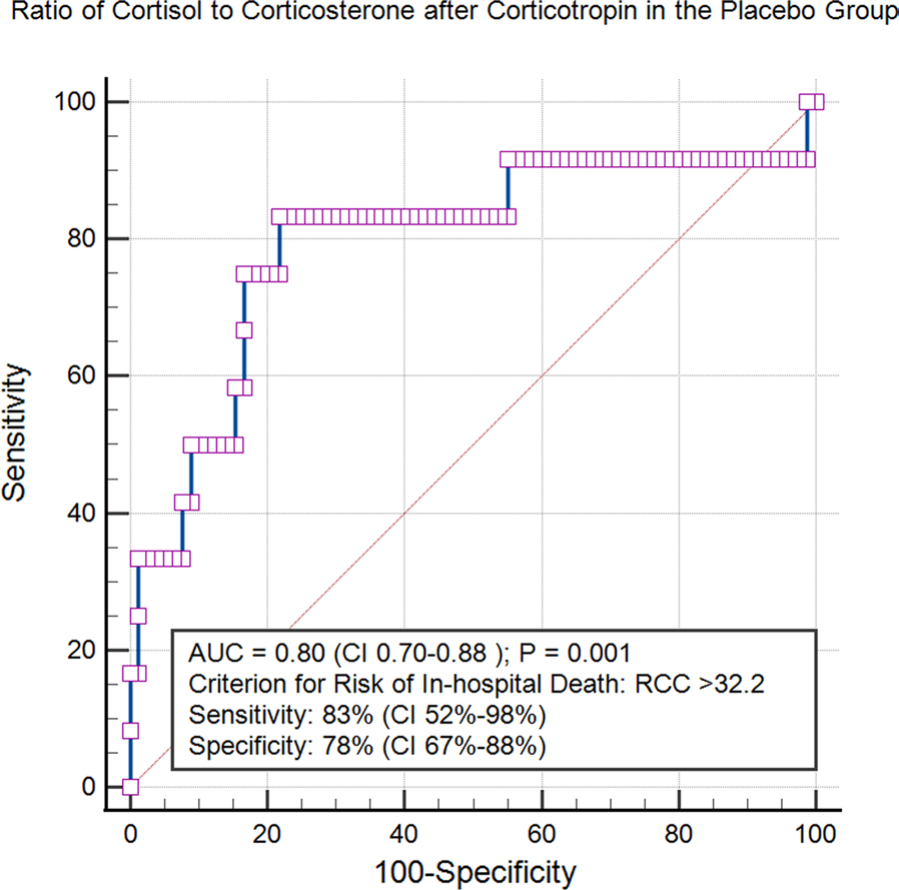
Receiver operating characteristic (ROC) curve for the ratio of cortisol to corticosterone (RCC) after stimulation corticotropin in the placebo group. A ratio greater than 32.2 was identified as a criterion for predicting in-hospital death

The ratio of cortisol to corticosterone (RCC) as a marker of gluco-mineralocorticoid balance in steroidogenesis showed in the ROC analysis an area under the curve of 0.80 (CI 0.70–0.88) with a sensitivity of 83% and a specificity of 78%. A ratio greater than 32.2 was found to be a good prognostic criterion for in-hospital death (Fig. 4).

Comparison of both ROC curves (corticosterone and RCC, both after corticotropin) showed no significant difference (difference between areas 0.7; 95% CI − 0.03 to 0.17; *p* = 0.18).

Of note, none of the healthy volunteers showed a RCC > 32.2, whereas 58 of 180 (32%) patients with sepsis showed this gluco-mineralocorticoid imbalance. The 12 patients in the placebo group who died in the hospital from sepsis showed a particularly pronounced imbalance (mean 62.1; 95% CI 32.4–91.8).

**Fig. 5:**
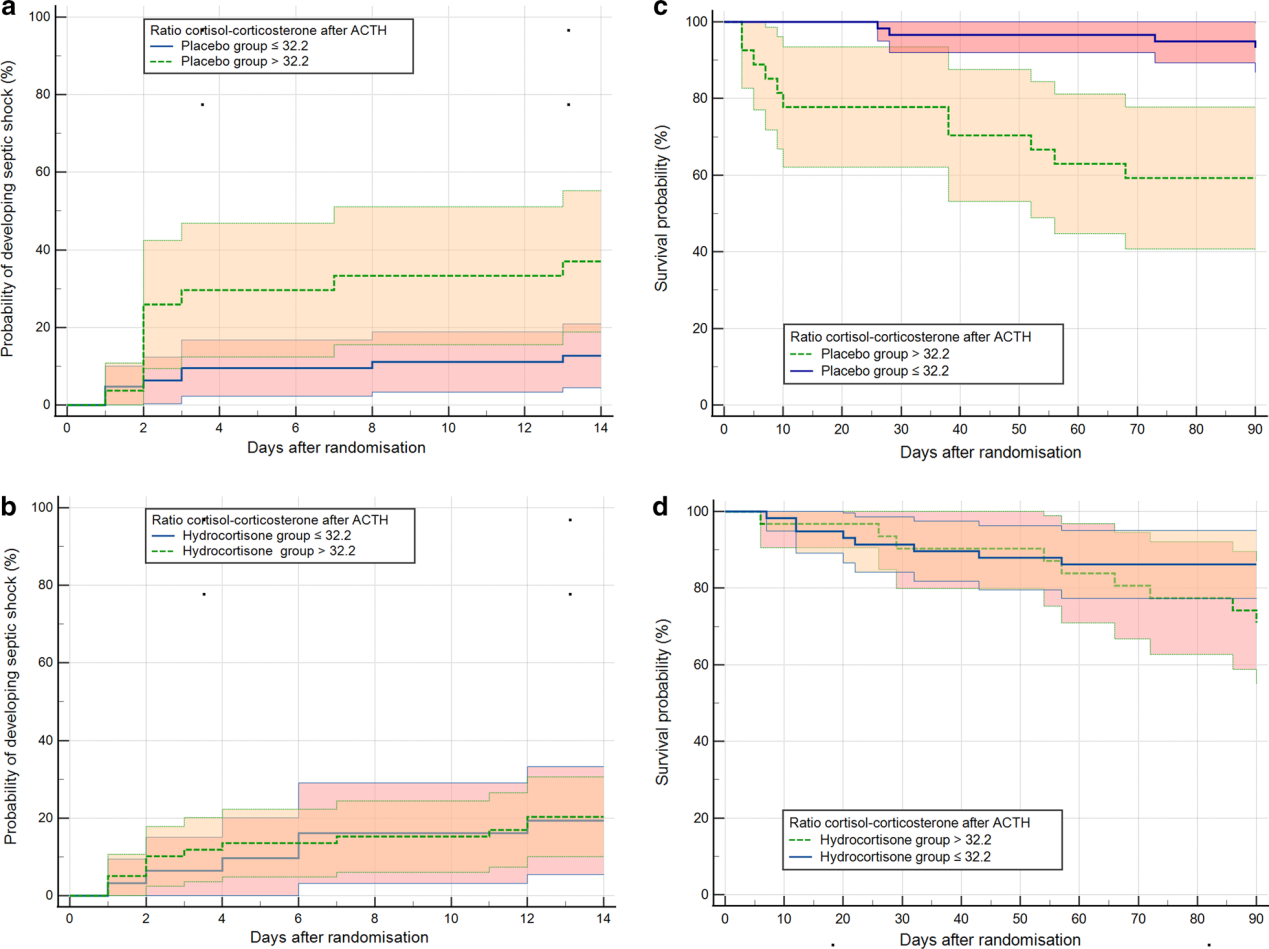
**A** and **B**, **C** and **D** Probabilities of septic shock development within 14 days (primary endpoint of HYPRESS) and 90-day survival (secondary endpoint) in the placebo and the hydrocortisone group as stratified by the ratio of cortisol–corticosterone greater or equal 32.2 and less (with 95% confidence interval for the curves). Patients in the placebo group with a cortisol–corticosterone ratio > 32.2 developed septic shock (*p* < 0.01) more frequently had a higher in-hospital mortality in the 90 days after randomization (*p* < 0.001). No differences were found in patients treated with hydrocortisone (200 mg for 5 days and a gradual dose reduction for another 6 days) stratified by the cortisol–corticosterone ratio

When this determined criterion of RCC is applied to the endpoints chosen in the HYPRESS trial, different survival curves result in the two treatment groups. In the placebo group, patients with an RCC <  = 32.2 rarely developed septic shock (13%) and had a low 90-day mortality (7%), whereas patients with an RCC > 32 developed septic shock significantly more often by day 14 developed (37%) (*p* < 0.01) and were significantly more likely to die by day 90 (41%) (*p* < 0.001). The RCC criterion of 32.2 showed no effect on the clinical endpoints studied in sepsis patients treated with hydrocortisone (Fig. 5A–D).

## Discussion

This exploratory analysis of glucocorticoid and mineralocorticoid steroids before and after stimulation with a corticotropin analogue yields well-known and novel results on adrenocortical function in sepsis. Both the glucocorticoid and the mineralocorticoid pathways are activated in sepsis, recognizable by the elevated 11-desoxycortisol and 11-desoxycorticosterone concentrations prior to corticotropin stimulation. Compared to healthy individuals, this leads to elevated cortisol but not corticosterone levels. After stimulation with corticotropin, the corticosterone response is more often attenuated in sepsis patients. Patients who died in the hospital show the lowest dynamic corticosterone response to corticotropin stimulation, which is significantly different from in-hospital survivors of sepsis. Excess cortisol over corticosterone after corticotropin stimulation is associated with increased risk of developing shock and death in patients not treated with hydrocortisone. This is not observed in patients treated with hydrocortisone.

A new finding in this exploratory analysis is an obvious impairment in mineralocorticoid steroidogenesis at the onset of sepsis. Although levels of 11-desoxycorticosterone (precursor of corticosterone) were elevated, this did not result in elevated corticosterone levels. Stimulation with corticotropin clearly unmasked this impediment to corticosterone synthesis despite high precursor levels. Corticosterone can be stimulated 4–15 times more by corticotropin in healthy humans compared to cortisol [12, 15, 19]. Conditions like sepsis appear to significantly impede its stimulability by corticotropin. It is noteworthy that 11ß-hydroxylase (see Fig. 1), which catalyzes the conversion of 11-desoxycorticosterone into corticosterone, is localized on the inner mitochondrial membrane [20, 21]. It is conceivable that mitochondrial dysfunction, as can typically occur in sepsis, hinders this enzymatic step [4, 22]. For example, the expression of steroidogenic acute regulatory protein (StAR) activity early in sepsis can affect this part of steroidogenesis by reducing the transport of cholesterol from the outer to the inner mitochondrial membrane [23, 24]. Unlike cortisol, these enzymatic steps appear to be essential for corticosterone.

In many animal species, corticosterone plays the central role in the stress response. In humans, this function falls to cortisol. The exact role of corticosterone in human physiology, other than its role as a precursor of aldosterone, is still unclear. Corticosterone has a higher affinity for the mineralocorticoid receptor (MR) than cortisol and can be detected in higher concentrations in cerebrospinal fluid than in serum [13]. Serial measurements of corticosterone and cortisol revealed that the pulsed secretion of both steroids is well synchronized with peaks in the morning [12]. Corticosterone metabolism is similar to cortisol with a slightly shorter half-life [19]. Corticosterone is released solely through corticotropin and serum levels at rest are very low. In stressful conditions, corticotropin pulses ensure a highly dynamic supply of corticosterone [12, 13].

In a canine model of *S. aureus* pneumonia-induced septic shock, the mineralocorticoid desoxycorticosterone (proximate precursor of corticosterone) decreased sepsis-induced organ dysfunctions, reversed shock, and improved survival but only when administered over 3 days prior to inoculation of *S. aureus*, but not after [25]. This suggests that activation of the mineralocorticoid signaling pathway exerts a protective effect. However, once septic shock has been induced, the administration of deoxycorticosterone no longer showed any effect in this model. This finding is compatible with a prophylactic but not therapeutic effect of the mineralocorticoid deoxycorticosterone [25].

The data of this study support the assumption that 11-deoxycorticosterone is no longer sufficiently converted into corticosterone in established sepsis in spite of high-dose corticotropin stimulation. This speaks for a hindered mineralocorticoid steroidogenesis. In this context, it is noteworthy that clinical trials that have so far been able to demonstrate an advantage for therapy with steroids in septic shock have always used the combination of hydrocortisone (predominantly a glucocorticoid) and fludrocortisone (a pure mineralocorticoid) both of which compensate for disturbances in the mineralocorticoid and glucocorticoid pathways [26, 27].

In this exploratory analysis, corticosterone and the cortisol-to-corticosterone ratio (CCR) were both identified as good predictors of clinical outcome after stimulation with corticotropin. CCR reflects the balance in activation of both, the glucocorticoid and the mineralocorticoid pathways. If the mineralocorticoid pathway was too compromised compared to activation of the glucocorticoid pathway, and thus, if the CCR was too high, this was associated with a worse clinical outcome.

Like cortisol, corticosterone secretion is suppressed by exogenous steroids like dexamethasone [12]. This underlines that corticosterone is dependent on corticotropin and may be compromised by any steroid treatment. For this reason, we separately analyzed the endpoints in the HYPRESS group randomized to hydrocortisone treatment. When using the criterion of cortisol-to-corticosterone ratio found in patients not treated with hydrocortisone, we saw a different evolution of study endpoints in patients treated with hydrocortisone. Glucocorticoid excess, as determined by the corticotropin-stimulated RCC > 32.2, did not predict the development of septic shock when patients were treated with hydrocortisone. A similar pattern was seen when looking at 90-day survival.

This exploratory study has several limitations. First, we measured only a subset of patients enrolled in the HYPRESS trial. Second, we cannot say what results steroid profiling will yield in patients with septic shock. HYPRESS deliberately recruited patients at the onset of sepsis without shock testing the hypothesis that with hydrocortisone the development of septic shock might be avoidable. Third, we did not measure aldosterone in short intervals, which would have been necessary due to the shorter half-life of aldosterone (approx. 20 min) to provide a better insight into the final pathway of the mineralocorticoid axis. It is quite conceivable that a deficiency in the precursor corticosterone can contribute to the aldosterone deficiency described in septic shock despite high renin activities [28].

The strengths of this exploratory study are that steroid profiling was performed using tandem mass spectrometry, which has a high analytical sensitivity and selectivity, and thus, the results are far less prone to be impaired by analytical problems of immunoassays [17, 29]. This does not preclude measuring the stimulated cortisol-to-corticosterone ratio with immunoassays in future. The ratio of two key steroids may be less affected by analytical problems in sepsis as both steroids have similar volume of distribution [3]. In addition, immunoassays of cortisol and corticosterone show low cross-reactivities [29]. In this way, the RCC combined with corticotropin testing may offer an innovative approach to revisit diagnostic challenges in adrenocortical function during sepsis [3, 17]. The stimulated RCC can possibly serve as an endocrine fingerprint for individualized steroid therapy in sepsis.

In conclusion, steroid profiling shows that the mineralocorticoid pathway is more frequently impaired than the glucocorticoid pathway in the stress response to sepsis. After corticotropin stimulation, patients with excess glucocorticoids over mineralocorticoids were more likely to develop septic shock and to die in hospital. The ratio of cortisol to corticosterone after stimulation with corticotropin can predict clinical endpoints such as shock development and mortality in sepsis and can be used to target hydrocortisone therapy. However, these hypotheses have yet to be prospectively validated in large cohorts of patients with sepsis or septic shock.

## Data Availability

Data may be made available in coordination with the SepNet Critical Care Trials Group, Jena, Germany.
